# Customisation’s impact on strengthening affective bonds and decision-making with socially assistive robots

**DOI:** 10.3389/frobt.2024.1384610

**Published:** 2024-10-14

**Authors:** Mohammed Shabaj Ahmed, Manuel Giuliani, Ute Leonards, Paul Bremner

**Affiliations:** ^1^ Bristol Robotics Laboratory, School of Engineering Mathematics and Technology, University of Bristol, Bristol, United Kingdom; ^2^ Faculty of Electrical Engineering, Kempten University of Applied Sciences, Kempten, Germany; ^3^ School of Psychological Science, University of Bristol, Bristol, United Kingdom; ^4^ Bristol Robotics Laboratory, College of Arts, Technology and Environment, University of the West of England, Bristol, United Kingdom

**Keywords:** customisation in robotics, personalised robots, affective bonds in HRI, trust in robotics, decision-making in HRI, socially assistive robots (SAR), human-robot interaction (HRI), persuasive robots

## Abstract

This study aims to fill a gap in understanding how customising robots can affect how humans interact with them, specifically regarding human decision-making and robot perception. The study focused on the robot’s ability to persuade participants to follow its suggestions within the Balloon Analogue Risk Task (BART), where participants were challenged to balance the risk of bursting a virtual balloon against the potential reward of inflating it further. A between-subjects design was used, involving 62 participants divided evenly between customised or non-customised robot conditions. Compliance, risk-taking, reaction time, and perceptions of the robot’s likability, intelligence, trustworthiness, and ownership were measured using quantitative and qualitative methods. The results showed that there were no significant differences in compliance or risk-taking behaviours between customised and non-customised robots. However, participants in the customised condition reported a significant increase in perceived ownership. Additionally, reaction times were longer in the customised condition, particularly for the “collect” suggestion. These results indicate that although customisation may not directly affect compliance or risk-taking, it enhances cognitive engagement and personal connection with robots. Regardless of customisation, the presence of a robot significantly influenced risk-taking behaviours, supporting theories of over-trust in robots and the automation bias. These findings highlight the importance of carefully considering ethical design and effective communication strategies when developing socially assistive robots to manage user trust and expectations, particularly in applications where behavioural influence is involved.

## 1 Introduction

In the fast-changing landscape of healthcare (Süssenbach et al., 2014) and education (Shiomi et al., 2015), the deployment of Socially Assistive Robots (SAR) symbolises a shift towards more interactive and personalised experiences. While SARs have demonstrated the potential to enhance outcomes and user experience, the efficacy of long-term robotic intervention critically hinges on understanding the factors that influence user engagement and trust. This study addresses a notable gap in Human-Robot Interaction (HRI) research, the role of customisation in SARs and its possible effects on enhancing the robot’s persuasive potential. This research aims to contribute empirical insights into designing and implementing strategies for more effective SAR deployments by examining the interplay between customisation and user perception in a structured experimental setting.

The success of SAR in promoting behaviour change depends on their persuasive power. In this context, persuasion refers to the robot’s ability to influence human opinions, attitudes or actions (Iyer and Sycara, 2019). According to Strack and Deutsch’s dual process theory, humans toggle between two modes of behaviour, reflective and automatic. Reflective behaviour, driven by the deliberative system, is rational and goal-directed. On the other hand, automatic behaviour involves reflexive reactions to our surroundings and situations, requiring little conscious thought. Conventional behaviour change interventions focus on reflective behaviour change, which aims to provide information to encourage people to change their behaviour (Colombo et al., 2007; Strack and Deutsch, 2004). However, psychological biases have shown that individuals have an altered perception of the objects they own or create themselves, which can affect their decision-making process. By inducing these affective biases, we aimed to investigate the persuasive potential of customising a robot that extends beyond conscious thought.

This study is based on the endowment effect (Kahneman et al., 1990), which suggests that people may perceive greater value in products they feel a sense of ownership over. The endowment effect highlights the increased value placed on objects solely due to ownership. Moreover, emotional engagement plays a crucial role in HRI, with design factors significantly impacting robot perception and the potential for affective bonds (Fong et al., 2003; Bartneck et al., 2009a). Combining these effects suggests that increasing psychological ownership through customisation can significantly alter people’s perceptions of the robot, thereby increasing its persuasive potential.

This study used a between-subject design where participants were randomly assigned to either the customisation or non-customisation conditions while interacting with a NAO robot during the Balloon Analogue Risk Task (BART) (Lejuez et al., 2002). The BART is a psychological task used to measure risk-taking behaviour. It involves the strategic inflation of a virtual balloon to accumulate points, with each subsequent inflation amplifying both the potential reward and the risk of bursting the balloon. The primary objective was to investigate the potential influences of the endowment (Kahneman et al., 1990) effect on decision-making and perception of the robot. This study engaged affective processes of trust (Lee and See, 2004), characterised by emotional closeness and empathy, within the context of robotics. By examining participants’ responses in the BART, we aimed to uncover how affective trust processes influence the interaction dynamics in HRI.

The main contribution of this paper is to explore how the non-functional customisation of a SAR, through the investment of time, energy, and creativity, can significantly alter user perceptions of the robot’s social agency and personality, thereby influencing decision-making processes. By investigating this dynamic, we aim to fill a notable gap in the current HRI literature, which often overlooks the psychological impacts of customisation. Specifically, we examine whether user customisation can enhance the robot’s persuasive potential in an unpredictable situation, providing empirical insights into the design and implementation of more effective SAR interventions. The theoretical basis for this work is the endowment effect, whereby increased psychological ownership should affect user interaction with SARs.

This paper is structured to offer a comprehensive exploration of the role of customisation within robotics. It begins with a review of relevant literature on the endowment effect, psychological ownership, and customisation in HRI. Next, we present the research questions and hypotheses being investigated. We then provide a detailed description of the method, including the between-subject study design, materials, procedure, and evaluation measures used to evaluate the robot’s influence and participants’ perceptions. The results section reports the main findings, followed by a discussion of the implications and limitations. Finally, conclusions are drawn, and future research directions are proposed to advance the understanding of how customisation may influence human decision-making and perceptions during HRI.

## 2 Related work

Psychological ownership, an important concept that is central to this study, is defined by Pierce et al. (2001) as the sense of possession and attachment one feels towards an object, influenced by factors like control, intimate knowledge, and personal investment of time, energy and creativity. This concept is important in HRI research, particularly in understanding how customisation and personalisation impact perception and engagement with SARs. Our literature review explores how customising a robot’s non-functional features to user preferences can psychologically influence their perception of it. This exploration is critical in examining whether such customisation enhances the robot’s perceived competence, warmth, and, ultimately, its persuasive potential in user interactions.

Users often personalise their robots by adding superficial accessories that don’t change the robot’s functions or interactions (Sung et al., 2009; Sauppé and Mutlu, 2015). Accessories have a long history of serving as a means for expressing identity, membership, belonging, social status, thoughts, and beliefs (Joshi et al., 2024). For example, primary school children co-designed an effective peer-tutor robot by adding a bow tie and buttons to enhance its appearance and playful qualities (Gena et al., 2020). Similarly, a virtual ethnographic study of the Pleo robot’s blogging community by Jacobsson (2009) showed that its users employed a diverse range of everyday clothing and fashion accessories to construct their own version of their robot’s social attributes and personality, expressing their individuality and bonding with it. The design and use of robot accessories suggest their significance not only for individual experiences but also for the broader construction of robots as social actors, as users attribute agency to them (Nass et al., 1994). This concept becomes particularly relevant in the context of HRI, where the degree of a robot’s customisation may influence how much it is perceived as “belonging” to a user and, thus, how readily a user might trust its suggestions based on accessory selection.

Further research examining the psychological implications of users customising, with customisation referring to users tailoring the robot’s features and capabilities to their preferences. Wang et al. (2016) and Delgosha and Hajiheydari (2021) indicated that user involvement in the customisation of robots and persuasive mobile health technology can significantly boost engagement, satisfaction, and a sense of ownership, enhancing user adoption and sustained usage, which is critical for inducing behavioural changes. In both cases, the endowment effect emerged, whereby people valued the objects more when contributing labour to their creation.

Reich-Stiebert et al. (2019) examined whether involving users in an educational robot’s prototyping and design process could positively change attitudes towards robots. In an online survey, they had students participate in prototyping an educational robot versus just evaluating an existing robot. Results showed that participation increased positive attitudes and reduced anxiety about educational robots. The findings suggest that involving end users in robot design can improve attitudes by making robots seem more familiar and realistic.

The ability to customise or personalise a robot’s appearance and behaviours has emerged as an important factor that can strengthen emotional bonds between humans and robots. Sung et al’s study of Roomba vacuum users provides compelling evidence that offering personalisation options facilitated feelings of attachment and commitment to the robot over months of use. Even though Roomba is not humanoid or intentionally designed for social connection, simple aesthetic alterations like decorative skins helped transform the device into a cherished family member for some owners. To support this, in the work of Delgosha and Hajiheydari (2021) and Lacroix et al. (2022), it was suggested that psychological ownership and trust could be fostered by the perceived control over and customisation of robots. These studies, however, point towards a possible saturation point beyond which additional customisation did not contribute significantly to these effects (Sung et al., 2009).

For example, Sung et al. (2007) found that personalisation and customisation of the robot led to higher perceptions of its competence and warmth. This is particularly relevant to our study’s focus on customisation’s potential to enhance a robot’s persuasive capabilities. Trust is a critical factor in this research and is defined by Lee and See (2004) as the belief in a robot’s ability to assist an individual in achieving their goals, especially in uncertain situations.

While customisation and personalisation have been shown to alter perceptions of robots, it remains to be seen whether psychological ownership translates into increased persuasiveness or compliance with a robot’s suggestion. An important question arises: Does customising a robot impact its perceived familiarity and warmth (attributes of trustworthiness), and therefore, how users perceive and interact with it? This complex intersection between customisation, perception, and interaction dynamics requires further exploration, and our study aims to address this critical gap.

In summary, endowment effect and psychological ownership suggest that feelings of ownership could increase a robot’s perceived value and persuasiveness. The current literature suggests potential strategies for cultivating more positive perceptions and acceptance of robots, which can be accomplished through facilitating personalisation, customisation, and co-creation. However, there is a need for additional research to directly investigate the persuasiveness and compliance in the context of robots. It is important to remember that while the deliberate design of customisation can result in robots that are better attuned to individual user needs, the ethical implications of increased attachment must be considered. Despite these concerns, well-implemented customisations could render robots more effective and likeable companions.

## 3 Hypotheses and research questions

The review of related work suggests that investing time, energy, and creativity in customising a robot may enhance the sense of ownership and perceptions of familiarity and warmth. Consequently, we hypothesise that customisation will impact decision-making processes (H1). To study H1, we aim to answer the following specific research question:

RQ1: To what extent does the customisation of a robot influence user decision-making and the robot’s perceived persuasiveness compared to a non-customised counterpart?

We will assess RQ1 by recording objective measures of persuasion to determine the extent of influence from robot intervention. We will record participants’ reaction time to make a decision after receiving a suggestion, as a faster reaction time is an indication of stronger persuasion. Additionally, the number of inflates after receiving a suggestion by the robot is recorded to determine if participants complied with the robot and to what degree.

The implications of user perceptions toward robots extend beyond mere compliance. The adoption of robotic systems, especially in sensitive sectors, hinges on user perceptions. Building on this, we hypothesise that customising a robot will foster a deeper sense of ownership and improve participants’ evaluations of the robot’s attributes (H2). To study H2, we aim to answer the following specific research question:

RQ2: How does the act of customising a robot’s appearance and features shape participants’ perceptions and evaluations of its attributes?

RQ2 will be methodically evaluated by capturing the subjective perceptions of the robot using questionnaires and detailed interviews. We want to determine whether there is a difference in how participants rate a robot on likability, intelligence, trustworthiness, and ownership scales when it is customised versus when it is non-customised.

## 4 Methods

The between-subject study was conducted in person, with semi-autonomous robot behaviour, to investigate the influence of robot customisation on persuasion and user perceptions during a Balloon Analogue Risk Task (BART). Participants interacted with a NAO robot across two BART blocks. The participants were informed that the robot would watch and learn their play style during the first game and provide suggestions based on their play style in the second game. However, in reality, the robot randomly suggested whether the participant should inflate the balloon or collect the points.

This study aimed to investigate if playing a game of chance with a customised robot leads to greater compliance with its suggestions than playing with a non-customised robot (RQ1). Additionally, we explored whether subjective perceptions towards the robot, including likability, intelligence, trustworthiness and perceived ownership, are affected by playing with a customised vs. non-customised robot (RQ2).

We utilised a mixed-methods approach that combined objective behavioural measures, validated questionnaire scales, and qualitative interviews to evaluate these research questions. This allowed us to comprehensively understand both the behavioural outcomes and user experiences related to robot customisation. The details of the study design, materials, and measures are provided in the sections below.

This study was approved by the Faculty of Engineering Ethics Committee at the University of the West of England, REF No: FET-2122-186. All data recorded were immediately anonymised; the only personal information collected was the participants’ age bracket and gender identification. Participants were assigned a unique, randomly generated four-digit code in case they wanted to remove their data within the grace period. Once participants completed the study, they were debriefed and told about the deception to ensure transparency.

### 4.1 Participants selection

Participants were recruited based on convenience sampling from the University of the West of England, the University of Bristol, and the Bristol Robotics Laboratory, which included students, staff, and visitors. All participants provided consent and received a £5 shopping voucher as a token of appreciation upon completing the study. To maintain the integrity of our results, we excluded pilot testers and individuals familiar with the primary researcher’s work from participating in the study.

### 4.2 Study design

#### 4.2.1 Task description

The BART is an established psychological task to assess risk-taking behaviour (Lejuez et al., 2002). It was chosen because it is a game of chance that focuses on repeatedly making choices with unpredictable outcomes. This aligns with our research interest in understanding the persuasive capabilities of robots in influencing decision making, as participants would have to decide whether the suggestion from the robot is trustworthy enough to follow.

The BART involved a game where participants strategically inflated a virtual balloon to accumulate points. Each inflation increased the potential reward and the risk of the balloon bursting. If the balloon burst, the participant lost all points for that balloon. To encourage participants to push their limits, they were instructed to achieve the highest score possible by maximising points while avoiding balloon bursts.

The study duration was approximately 30 min, comprising two blocks of the BART; each block contained 30 balloon trials. Each balloon per trial had a randomly assigned inflation limit. These limits were spread across the 30 balloon trials in each block and adhered to a normal distribution with a mean set at 16 inflations and a standard deviation of 5. To maintain consistency with prior research (Lejuez et al., 2002), the maximum limit for any balloon was capped at 32 inflations.

#### 4.2.2 Procedure

To start, the researcher briefed each participant on what they would do during the study; after this, the researcher moved behind the separating wall to allow the participants to complete the study independently. The user interface gave the participant a consent form detailing the ethical considerations taken and how the data from this study would be used.

After this, all participants were asked to customise a robot. All participants then experienced a simulated “lost connection” error on the screen where the robot would appear to shut down. Depending on the condition the participant was assigned, they would either get to keep the robot (Customisation condition) or have the robot replaced with its default configuration (Non-customisation condition). To give both groups the same impression of reliability, all participants experienced the shutdown error.

Participants were then given a detailed description of what they and the robot would do for each of the two blocks of the BART before starting. In the description, the robot was framed as a companion that learned the participant’s gameplay style so that it could help them in the second (robot-assisted) block. The participant got acquainted with the task in the first (baseline) block of the BART. After completing both blocks, participants answered survey questions followed by a face-to-face interview with the lead researcher.

We used this between-subjects study design as we were confident that it ensured that the observed differences between groups would more accurately be attributed to robot customisation, enhancing the representativeness of the results.

After detailing the initial setup and the procedure undertaken by participants, we turn our attention to the main elements of our study that affect our research questions: the customisation process, the transition narrative, and the specifics of robot interaction. These components are crucial in understanding how the personalisation of a robot influences participant behaviour and perceptions.

#### 4.2.3 Customisation

**FIGURE 1:**
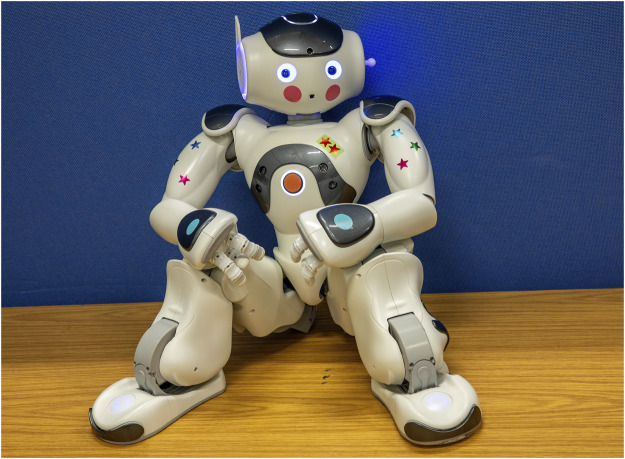
Example of a robot customised by a participant, showcasing personalised features such as LED colour changes, 3D-printed accessories attached to the head, and stickers around the body.

**FIGURE 2:**
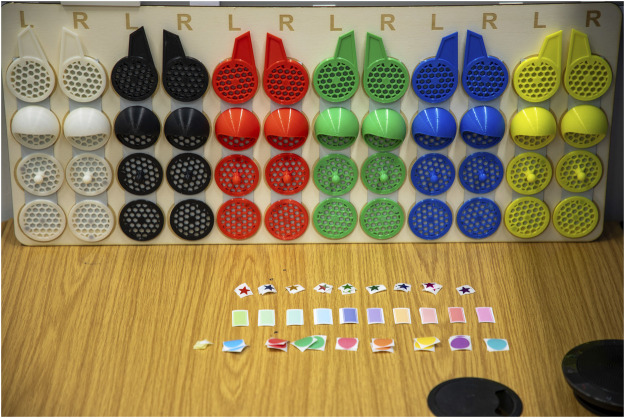
Physical customisation options provided to participants, showing 3D-printed parts for the robot’s head and stickers for its body.

The customisation portion of our study was designed to assess whether personalising a robot would impact participant compliance and their subjective perceptions of the robot. To ensure consistency across study conditions, we standardised this process, allowing participants to tailor the robot to their preferences through a series of steps. For visual reference, Figure 1 showcases the available customisation options, while Figure 2 displays an example of a robot customised by a participant. The customisation process included:

1. Naming the robot
2. Altering the robot’s voice pitch
3. Altering the robot’s voice speed
4. Changing robot LED’s colour
5. Attaching parts to the side of the robot head
6. Attaching stickers to the body of the robot

These options were chosen after evaluating all possible non-functional customisation options available on the NAO robot and were approved by pilot testing. Previous literature (Delgosha and Hajiheydari, 2021; Lacroix et al., 2022) has suggested that the customisation options should not be too elaborate, as they can have the opposite effect of what is intended.

We framed this part of the study as making the game more engaging. The user interface guided the participant through the customisation options. There were two pages of customisation; on the first page, options to change the robot’s settings (1, 2, and 3) were made available, and on the second page, options to change the appearance of the robot (3, 4, and 5) were made available.

#### 4.2.4 Transition narrative

When designing a study, we need to take into consideration the confounding variables that may affect it. From pilot testing, we identified that interaction with the robot during the customisation stage made participants favour the robot more. Therefore, to keep the study fair, we ensured that the same degree of interaction with the robot was maintained in both groups. This way, we could isolate the effect of customisation on decision-making processes.

To transition participants into the main experimental condition without disrupting the flow of the study, we devised a carefully crafted narrative. After customising the robot, participants were shown a simulated “lost connection” error message, which was crucial for two reasons. Firstly, it allowed us to switch the robots between the customised and non-customised conditions seamlessly; secondly, it maintained the integrity of the participant’s experience, ensuring that the level of engagement with the robot across study conditions remained consistent throughout the study.

Before the study, participants were informed that the system might be unreliable. Four seconds after the game introduction page loaded, the robot simulated a “lost connection” error, and a shutdown procedure would be initiated, displaying an error message on the screen. At this point, the non-customisation group would have their robot replaced with a new robot that had a default setup. This new robot would introduce itself with the name “NAO” before starting the game. The customisation group, however, would have their robot regain connection and proceed with the study.

By employing structured components like customisation and a transition narrative, we ensured that each participant’s experience was consistent throughout the study, with the only difference being whether the robot was customised or not. This allowed us to isolate the influence of customisation on decision-making.

#### 4.2.5 Interaction mechanics

The interaction mechanics between the participants and the robot during the BART gameplay were carefully designed to evaluate the persuasive power of the robot across both customised and non-customised conditions.

**FIGURE 3:**
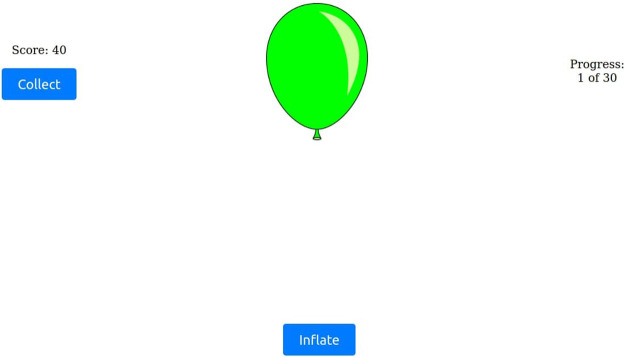
Screenshot of the main BART gameplay, where participants collect points by pressing the “collect” button.

**FIGURE 4:**
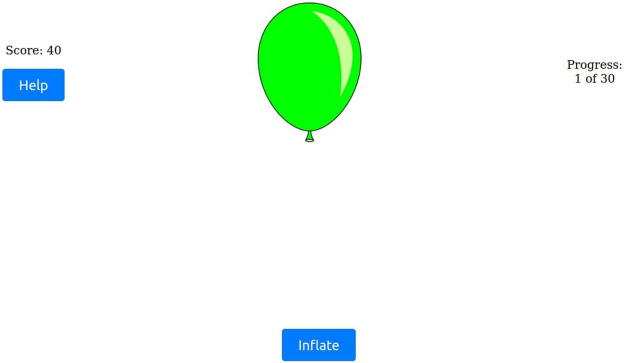
Screenshot of the second BART block, where participants must press “Help” before collecting points.

A key feature of the user interface was the adaptation of the game interface in the second BART block. The “Collect” button label, as depicted in Figure 3, was replaced with the “Help” button, as illustrated in Figure 4. Pressing “Help” prompted the robot to offer a suggestion, simultaneously reverting the button label to “Collect”, thus allowing participants to decide whether to act on the robot’s advice.

To standardise the experimental conditions, each participant was allowed to request help only once per trial before being allowed to collect their points. This setup provided a consistent measure of each participant’s decision-making process after receiving a suggestion.

The study comprised two blocks of BART gameplay. The first block served as a calibration phase, without robot assistance, helping participants familiarise themselves with the task and establish their personal risk tolerance level. During the second block, where the robot provided a suggestion, we assumed that the participant would request help at a critical decision point, specifically when they believed further inflation would burst the balloon. At this point, the suggestion from the robot to inflate further or collect the points would be accompanied by a significant risk, as perceived by the participant. Therefore, the decision to proceed would be a direct measure of the participant’s trust in the robot’s suggestion. Evidence supporting these assumptions, based on the data we collected, is provided in the supplementary material accompanying this paper.

The robots’ suggestion to inflate or collect points was pseudorandomised, with equal probability for either suggestion across the 30 trials. This pseudorandomised sequence was generated for each participant. This approach was essential for ensuring that the robot’s influence on participant decisions was not predictable, thereby maintaining the integrity of the study.

Given the random nature of the feedback, it was inevitable for the robot to give a suggestion that would burst the balloon. Therefore, framing of the robot was essential. In line with previous research, Paepcke and Takayama (2010) suggests deliberately setting lower expectations for robot performance reduces disappointment when the robot does not meet its expectations. Therefore, we were careful not to position the robot as an expert but instead as a partner learning how to play the game with the participant.

The robot was positioned to the participant’s right and remained seated throughout the study 6. The robot faced the participant in the customisation phase and faced the screen during the gameplay. When providing suggestions, the robot employed a combination of verbal and non-verbal communication. Social agency theory states that the more social cues an artificial agent uses, the more it activates human social interaction schemata, making it more persuasive (Mayer et al., 2003). The response for inflation was always one nod accompanied by a suggestion, “I would inflate the balloon.” The response for the collection of points was shaking its head once accompanied by the suggestion, “I would not inflate the balloon.”

#### 4.2.6 Materials/equipment/setup

**FIGURE 5:**
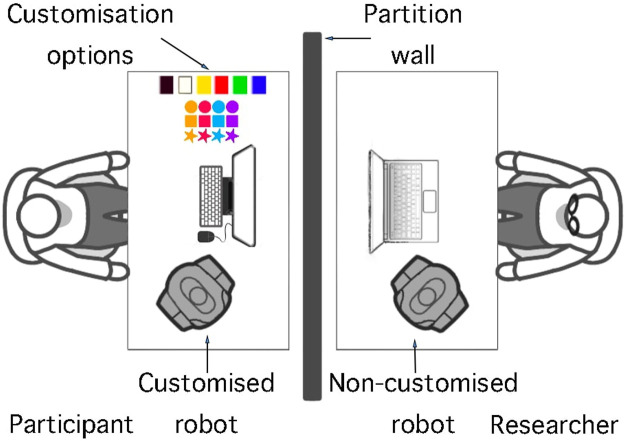
Artwork illustrating the study setup, showing how participants were isolated from the researcher during the study. On-screen instructions were provided, with the researcher intervening only to reconnect or swap robots depending on the participant’s study condition.

**FIGURE 6:**
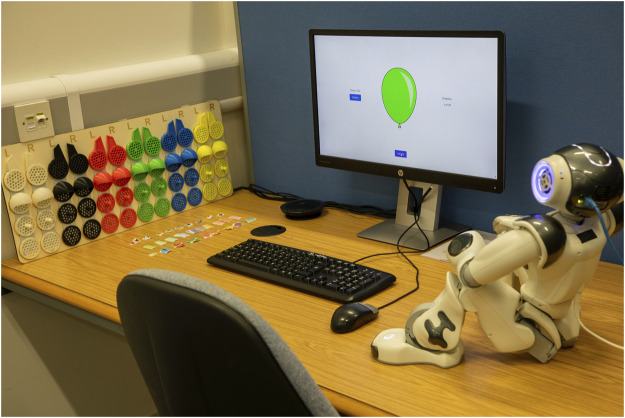
Photo of the actual study setup. Customisation options are on the left, with the robot seated on the right. On-screen instructions were provided, and text-to-speech accessibility was enabled via a speaker placed beneath the monitor.

For an illustration of the study setup, please refer to Figures 5, 6. The study was conducted in an open-plan area divided by movable walls. The participant and the researcher were on opposite sides, separated by a partition wall. On the participant’s side, they interacted with a monitor, keyboard, mouse, physical customisation options, and a NAO robot. Two NAO robots were used during the study, one for each study condition. The researcher’s laptop was connected to the participants’ monitor, with the screen being mirrored; this was so the researcher could identify which portion of the study the participants were in and intervene if an error occurred.

### 4.3 Data collection and processing

We adopted a mixed-methods data collection approach to comprehensively understand participants’ behaviour and experiences throughout the study. Both qualitative and quantitative measures were used to enhance the reliability and validity of the data.

Objective behavioural measures (4.3.1) were used to quantitatively assess participants’ compliance with the robot’s suggestions, while validated questionnaire scales assessed subjective perceptions of the robot. The subjective questionnaires were administered once all blocks were completed, and participants could share feedback in a free-text format. Finally, the study was concluded with a face-to-face interview with the lead researcher.

Our dual focus was on quantifying metrics, such as how often participants followed the robot’s recommendations (RQ1) and capturing participants’ nuanced, subjective experiences during the task and their interactions with the robot (RQ2).

Combining these measures, we aimed to offer a holistic view of how participants interacted with the robot and how its features may have impacted their decision-making in the task.

#### 4.3.1 Objective measures

We recorded specific metrics to assess compliance with the robot’s suggestions; these included the robot’s recommendation, the subsequent action taken by participants (specifically, whether they chose to inflate the balloon after receiving the robot’s recommendation), the number of inflates made after the robot provided its suggestion, the inflation limit for the balloon to ensure it did not burst, and the time it took to make a decision after they requested help from the robot.

#### 4.3.2 Subjective measures

To evaluate the quality of the human-robot interaction, we utilised previously validated multi-item scales, with participant responses recorded on a 7-point Likert scale. All scales were adjusted according to the context of human-robot interaction and to align with the research question being investigated. The scales used included:

• Godspeed-Likability and Godspeed-Intelligence Scales: These established scales were used to measure participants’ perceptions of the robot’s likability and intelligence (Bartneck et al., 2009b).

• Psychological Ownership: Adapted from Van Dyne and Pierce (2004), to assess the degree to which participants felt a sense of ownership or connection with the robot.

• Trustworthiness Questionnaire: There is no universally accepted measure of trust in robotics; therefore, we chose the most applicable one. Adapted from Jian et al. (2000), to assess participants’ trust in the robot’s suggestions and actions.

#### 4.3.3 Qualitative insights

Post-study interviews were conducted for each participant and were transcribed in person using speech-to-text software to supplement our quantitative data to gather in-depth insights into participants’ experiences and perceptions. These questions served as a manipulation check designed to elicit responses related to the research questions.

### 4.4 Data analysis

This section provides a detailed breakdown of the various tests and analyses performed on the collected data, including Likert scale questionnaires, structured face-to-face interviews, and objective behavioural measures, ensuring a transparent and comprehensive insight into the analytical process.

#### 4.4.1 Data processing

Our analysis excluded data from the first six balloons as we assumed participants were acclimating to the game and the robot during these initial balloon trials. This approach helped in mitigating potential learning effects on the data.

Data processing were categorised into three main areas:

1. Compliance with robot suggestions

• we monitored the actions taken after help was requested. We established that compliance for an inflate request was achieved when the participant pressed inflate after requesting help, and for a collect request, compliance was achieved when the participant pressed the collect button after requesting help. Percentage compliance was calculated as the total number of times the participant complied with the robot’s suggestion divided by the total number of times they asked for help from the robot.

• The number of inflates after help was requested was also recorded. This was used to indicate the level of trust ascribed to the robot by how much the participant inflated the balloon after requesting help. From this, the average inflates after help was requested was calculated by dividing the sum of inflates after help requests by the number of times help was requested.
2. Risk-taking behaviour during the experiment, we measured the total balloon inflation, which refers to the number of times a participant pressed the inflate button for each balloon. From this, we calculated the average balloon inflation across both blocks by dividing the sum of the total inflates by the number of balloons that did not burst in the given block. This metric assessed any differences in participants’ behaviour across the study conditions and across both games regardless of the study conditions.
3. Reaction time to decide what to do after the request help button was clicked was recorded. Initially, we normalised these data using the mean reaction time data; however, due to outliers skewing the data, we decided to use the median reaction time to reduce the impact of outliers on the data analysis.

Additionally, the exclusion criteria for objective data were set to two standard deviations from the mean. Two standard deviations capture 95% of the dataset; therefore, it was assumed that any data outside of this bound must be an outlier. Each of the above categories was treated as independent observation; the absence of excluded participants in multiple datasets supports this assumption.

For the Likert-scale questionnaire, we computed the mean score for each scale to gauge the general perceptions of the robot among the participants and if this changed between the groups. The mean for each questionnaire was used since the Likert scale is analysed as interval data; therefore, the mean is used to find the central tendency for each participant.

#### 4.4.2 Analytic methods

Objective datasets were assessed using Shapiro-Wilk normality and Levene’s homogeneity tests post-exclusion criteria. To ensure the accuracy of the Shapiro-Wilk normality test, the Q-Q plot was examined for the impact of outliers, as the test is sensitive to small sample sizes. Non-parametric methods were used for datasets that did not meet these assumptions.

To compare independent observations, the Wilcoxon rank-sum test, the non-parametric alternative of the independent samples t-test, was used, and the test results were based on an approximation method due to the presence of ties. We used this test to evaluate the compliance analysis for inflation behaviour after receiving help from the robot.

To compare related observations for non-group comparisons, the paired samples t-test was used. In cases where the data violated the assumptions of parametric tests, the Wilcoxon-signed rank test was used. We analysed compliance and risk-taking behaviours for non-group effects.

**FIGURE 7:**
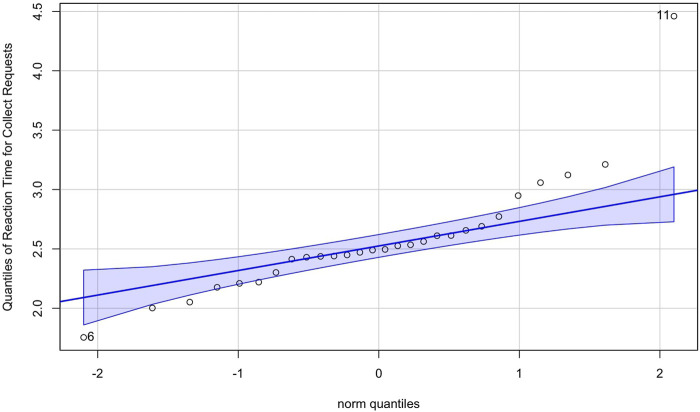
Q-Q plot of reaction times following a “collect” request, indicating a near-normal distribution except for a single outlier affecting the Shapiro-Wilk test.

The reaction time data were evaluated for parametric testing; however, one of the data subsets did not satisfy the assumptions of parametric testing; however, after reviewing the Q-Q plot of the distribution, it was observed a single outlier was significantly impacting the Shapiro-Wilks normality test, see Figure 7. Therefore, we decided to assume the data satisfied the assumptions of parametric testing, and we used mixed ANOVA to evaluate the impact of the robot’s response on participant reaction time across both study conditions.

In using non-parametric Tests, we acknowledged the potential limitation of reduced statistical power, particularly with smaller sample sizes. We supplemented significant results with *post hoc* analyses and effect size. This was to ensure significant findings were noticed and provide a measure of the practical significance of our results.

As for the subjective questionnaires, Shapiro-Wilk and Levene’s tests confirmed the normal distribution (p>0.05) and homogeneity of variances (p>0.05) for likeability, intelligence, trustworthiness, and ownership metrics, leading to the use of independent samples t-tests. Effect sizes were also calculated to understand the observed differences’ practical significance.

The structured interview was analysed quantitatively for closed-ended questions, while open-ended questions were analysed thematically. Themes related to the participant’s perception and reception of robot suggestions were identified to understand the subjective aspects of robot and participant interaction. Percentage analysis was carried out to represent the participant’s responses numerically. The themes identified were related to robot intelligence, helpfulness, and ownership.

## 5 Results

The final analysis included 62 participants (43 male, 19 female) with ages distributed as follows: 18–25 (19 participants), 26–35 (29), 36–45 (8), 46–55 (3), and 56+ (3). Participants were evenly distributed across study conditions, with 31 participants in each group. The exclusion criteria for each objective analysis was two standard deviations from the mean, and the number of participants included in the analysis is specified in the appropriate section. No participants were excluded from the subjective questionnaire analysis, as there were no clear predefined criteria for exclusion. The significance level was set at α=0.05 .

To maintain the readability of the main body of the paper, we have included additional supporting data in the Supplementary Material, intended for readers who are interested in a deeper understanding of the data.

### 5.1 Objective data

#### 5.1.1 Compliance

**FIGURE 8:**
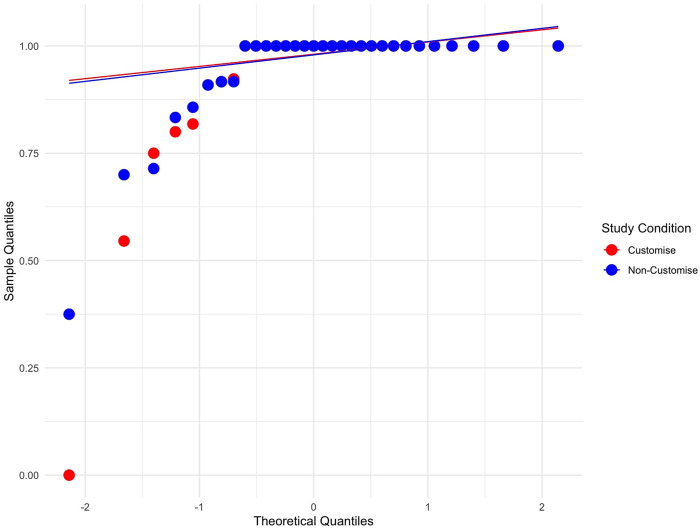
Q-Q plot showing normalised compliance with the robot’s suggestion to inflate the balloon across both study conditions. The non-normal distribution and ties made traditional statistical analysis unsuitable for comparison between conditions.

**FIGURE 9:**
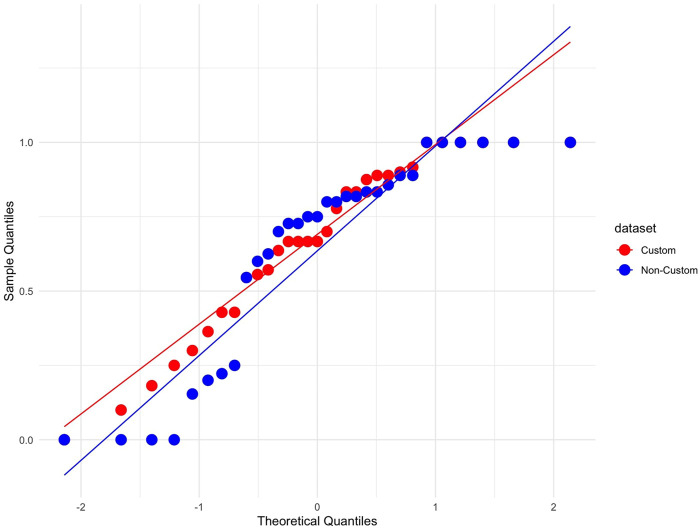
Q-Q plot showing normalised compliance with the robot’s suggestion to collect points across both study conditions. The non-normal distribution and ties made traditional statistical analysis unsuitable for comparison between conditions.

**FIGURE 10:**
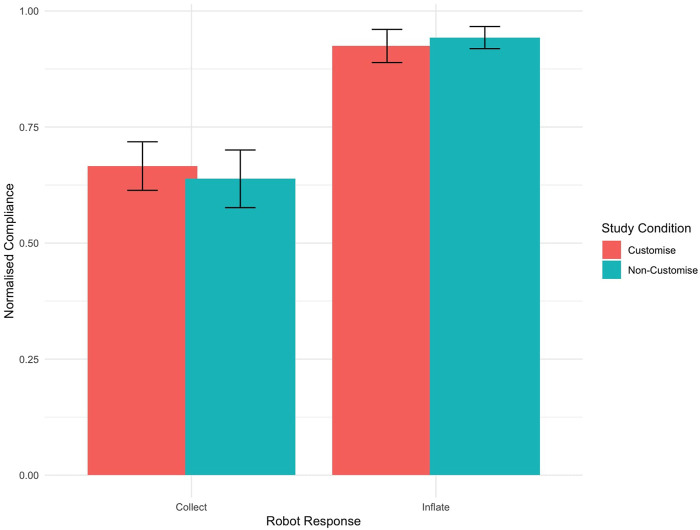
Bar graph showing participant compliance behaviour in response to robot suggestions. The graph compares compliance rates across different study conditions. Error bars indicate the Standard Error of the Mean, reflecting the variability and precision of these averages.

The data were analysed to evaluate the percentage of compliance with the suggestions made by the robots. The dataset was split into compliance percentages for each robot response (“inflate” and “collect”) between study conditions. The analysis of these subsets showed little variation between the participants, as depicted in Figures 8, 9. Due to the non-normal distribution and the prevalence of ties within the data, traditional statistical analysis was deemed inappropriate for comparisons between study conditions. Instead, a visual inspection was conducted, leveraging the graphical representations in Figures 8–10. This visual assessment suggested that participants in both the customised and non-customised conditions showed comparable compliance percentages for each robot suggestion, indicating that robot customisation had no significant impact on participants’ adherence to robot requests.

Although there were no significant differences between the study conditions, the compliance behaviour within study conditions for “inflate” and “collect” suggestions was visually different. This data set satisfied the requirement for no-parametric tests. As a result, the Wilcoxon Signed-Rank Test demonstrated a significant effect of robot suggestion on participant compliance behaviour ( $p=1.369e^{-7}$ , effect size r=0.704 ). This suggests a strong influence of robot suggestions on participant decision on whether to inflate the balloon or collect the points, as shown in Figure 10.

To further understand the trust participants placed in the robot’s suggestions, we analysed their inflation actions after receiving a suggestion. In this analysis, 56 participants passed the exclusion criteria. The Wilcoxon Rank Sum test showed no discernible difference in participants’ inflation behaviour between the study conditions after receiving a suggestion (p=0.598) , indicating that the customisation did not significantly impact decision-making processes related to inflation.

**FIGURE 11:**
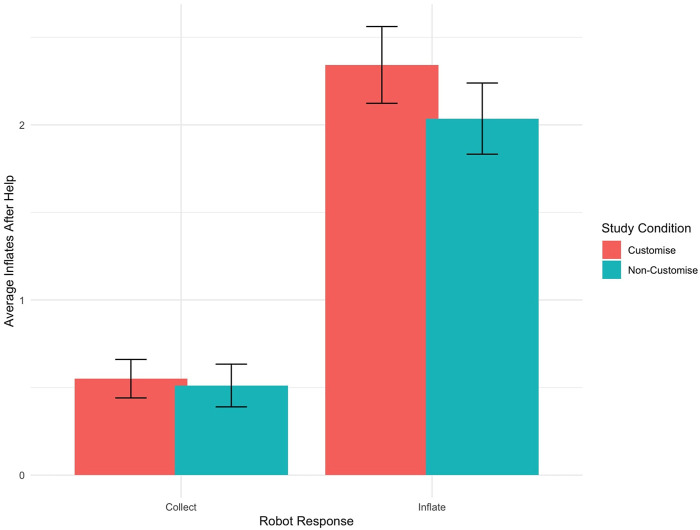
Bar graph showing the average number of inflations following “inflate” and “collect” requests by robots across different study conditions. Error bars indicate the Standard Error of the Mean, reflecting the variability and precision of these averages.

To gain deeper insights, the Wilcoxon Signed-Rank Test assessed the direct impact of robot requests (“inflate” versus “collect”) on inflation behaviour, regardless of the study condition. The analysis indicates a significant influence of robot requests on inflation behaviour after receiving a request ( $p=1.816e^{-10}$ , effect size r=0.858 ), as depicted in Figure 11. Participants inflated the balloon more times when the robot requested an “inflate” and less when the robot requested a “collect.” This shows that the participants trusted the robot’s suggestion, even though it was introduced as a learning companion and, therefore, didn’t have knowledge of the consequences of its suggestions.

### 5.2 Risk taking behaviour

A total of 56 participants passed the exclusion criteria for inflation behaviour. The Wilcoxon Rank Sum test found no significant difference in how much participants inflated the balloon between study conditions (p=0.150) , indicating that customising the robot did not influence participants’ propensity to take risks.

**FIGURE 12:**
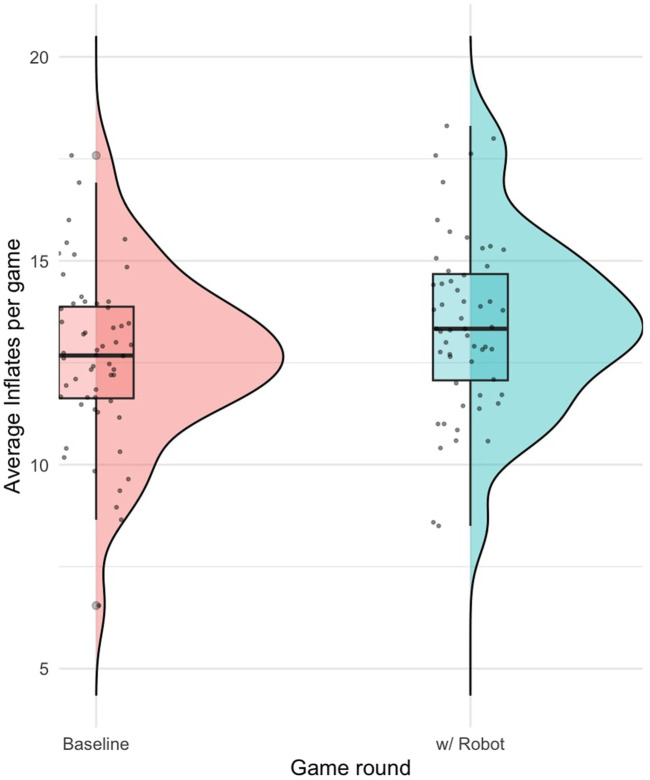
Distribution of average inflations per game, comparing the baseline (first BART block) to the robot-assisted condition (second BART block).

A paired samples t-test comparing the average inflation for each balloon against the baseline game revealed a significant difference $\left(p=1.8e^{-4}\right)$ with a moderate effect size $\left(Cohen^{\prime }sd=0.542\right)$ . This suggests a noticeable change in risk-taking behaviour in the robot’s presence, as shown in Figure 12.

#### 5.2.1 Reaction time

**FIGURE 13:**
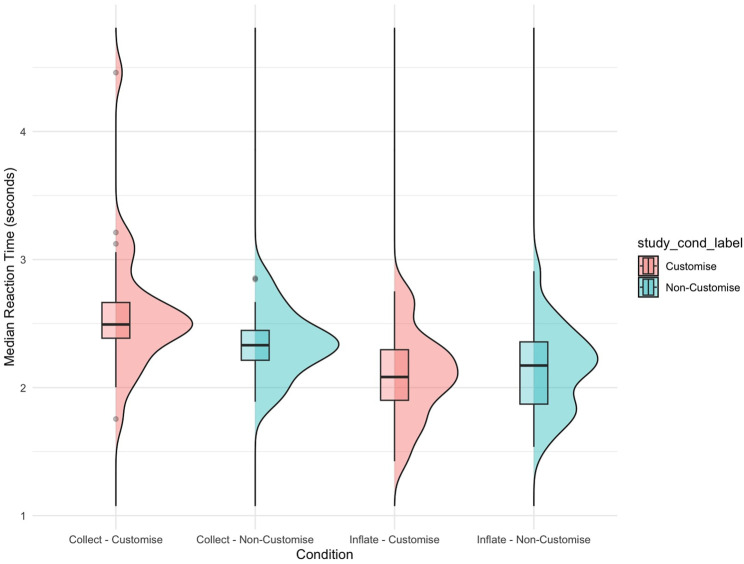
Box plot and distribution illustrating differences in reaction time following “collect” and “inflate” suggestions by the robot in customised and non-customised groups. Significance is observed in the ‘collect’ request between the two study conditions.

In assessing how robot suggestions influenced participant reaction times, our analysis included data from 56 participants who met the inclusion criteria. A Mixed ANOVA revealed a significant interaction effect between the study condition (customised vs. non-customised robot) and the type of robot suggestion (inflate vs. collect) $\left(F\left(1,53\right)=7.151,\;p=0.01,\eta^{2}=0.038,Cohen^{\prime }sf=0.199\right)$ , indicating a moderate influence of the robot’s suggestion on reaction time. This effect was primarily driven by the “collect” response (p.adj=0.031) ; no significant differences in study conditions were observed for the “inflate” response (p.adj=0.583) . This finding suggests that the interaction between the type of robot suggestion and the customisation condition significantly influences participants’ reaction times, especially in scenarios involving the “collect” response where the time it took to decide was extended, as shown in Figure 13.

In addition, there was a significant main effect of robot response $\left(F\left(1,53\right)=41.756,p<3.07e^{-8},\eta^{2}=0.185,Cohen^{\prime }sf=0.476\right)$ . The results indicate a considerable influence of the robot suggestion, whether to “collect” or “inflate,” independent of study conditions, on reaction time. Pairwise t-test comparisons within each study condition confirmed significant differences in reaction times for both non-customisation (p.adj = $5.88e^{-4}$ ) and customisation (p.adj = $1.65e^{-5}$ ) conditions. These results highlight the significant impact of robot response on decision-making times, irrespective of the study condition. Participants took longer to decide to collect points than to decide on inflating.

### 5.3 Subjective data

Independent samples t-tests found no significant differences in perceptions of likability (t(60)=−0.162,p=0.872) , intelligence (t(60)=−0.102,p=0.919) , or trustworthiness (t(60)=0.086,p=0.932) between the customised and non-customised conditions.

The t-test showed that feelings of ownership were significantly higher for the customised robot than for the non-customised robot $\left(t\left(60\right)=-3.057,\;p=0.003,Cohen^{\prime }sd=0.776\right)$ . The average ownership score for the customise condition was 3.968 (SD = 1.516), with a median of 4.0 and an interquartile range of 1.667. In contrast, the non-customise condition had an average score of 2.849 (SD = 1.360), a median of 2.667, and an interquartile range (IQR) of 2.0.

In the concluding interview, it was found that 61% of participants felt influenced by the robots, while 79% found them helpful, with 8% finding it partially helpful. Moreover, out of all the respondents, 42% believed that the robot’s prediction was accurate, with 27% thinking it was partially accurate, 19% did not, and 11% were unsure. Furthermore, 30.95% of the participants said they would like the robot that provided the correct suggestion, regardless of whether they customised it.

There were little to no group differences, except when participants were asked if they felt a sense of ownership over the robot. 21% of the participants felt a sense of ownership of the robots. However, in the customisation group, 31% felt a sense of ownership, while in the non-customisation group, this was only 9%.

### 5.4 Summary

This study investigated the effects of customisation on user interactions with SARs, particularly examining compliance, risk-taking behaviours, reaction times, and subjective perceptions of ownership, likability, intelligence, and trustworthiness.

1. Compliance and Risk-Taking Behaviors: Our findings revealed no significant differences in compliance or risk-taking behaviours between participants in the customised and non-customised robot conditions.
2. Reaction Time: Interestingly, customisation affected reaction times, specifically in scenarios involving the robot’s “collect” suggestion.
3. Subjective Perceptions: Among the subjective perceptions measured, only feelings of ownership were significantly higher for participants interacting with customised robots than those with non-customised robots.
4. Trust in Robot Suggestions: Despite the lack of significant differences in compliance and risk-taking based on customisation, the study found that robot suggestions significantly influenced participants’ decision-making. This effect underscores the inherent persuasive power of SARs’ suggestions, irrespective of customisation.

These findings suggest that while the study condition does not drastically change behaviour, it can subtly influence certain aspects of decision-making and foster a personal connection to the robot. This reveals the subtle influence of customisation in enhancing user experience with SARs. These findings provide a basis for further discussion of the practical implications and potential opportunities to improve the effectiveness of SARs in real-world applications.

## 6 Discussion

This study explored the effects of robot customisation on human decision-making, focusing on compliance, risk-taking behaviour, reaction time, and robot perceptions. Our findings offer nuanced insights into these dynamics, contributing to the broader understanding of human-robot interaction, challenging some assumptions and affirming others.

### 6.1 Compliance

Our investigation found that customising a robot did not significantly impact compliance, which contradicts our hypothesis (H1) that participants would be more compliant with a robot they had customised. This suggests that customisation did not increase the persuasiveness of the robot.

Significant effects of robot response on participants' compliance and trust were independent of customisation. Participants trusted the robot’s suggestion and adjusted their gameplay response based on the robot’s suggestion by inflating the balloon more times for an inflate request than a collect request, even though they knew that the robot did not have more information than them about the game. Our findings align with previous studies, such as Robinette et al. (2016), showing that people trusted a robot in an emergency exit navigation task even though it showed that it was poor at navigation. The findings indicate a general trust in the robot’s suggestions, which aligns with previous research showing that people tend to trust automated systems. This trust may be due to an automation bias. Hence, customisation may not strongly influence the general trust in robotic systems, challenging the assumption that customisation enhances compliance.

### 6.2 Risk taking

Our study found no significant difference in risk-taking behaviour between customised and non-customised robot conditions. However, participants exhibited increased risk-taking when playing the game with the robot, as compared to playing it on their own. There was no difference in increased risk-taking due to customisation. This suggests that the mere presence of a robot, regardless of its customisation, greatly influences participants’ willingness to take risks. However, this finding needs to be interpreted cautiously as previous studies (De Groot, 2020) have demonstrated that past experiences with the BART can lead to increased risk-taking. Although in general, this finding is crucial for the ethical design and implementation of robotic systems, especially in decision-critical contexts. It underscores the need for careful consideration of how robot behaviours and suggestions can impact human actions, regardless of customisation.

### 6.3 Reaction time

The analysis of reaction times following robot suggestions demonstrated a significant influence of both the type of suggestion and the study condition on participants’ decision-making speeds. The primary finding was that reaction times varied significantly between the “inflate” and “collect” suggestions within each study condition. This difference can be attributed to the nature of the “collect” decision, which carries more weight as it is a one-time decision, in contrast to the “inflate” decision that allows for continued point accumulation.

Further analysis revealed that the interaction effect between study conditions (customised vs. non-customised robot) and the type of robot suggestion was significant, particularly for the “collect” response. Participants in the customised robot condition took longer to respond to a “collect” suggestion compared to those in the non-customised condition. This indicates a more deliberate and possibly more careful decision-making process when participants interacted with a customised robot.

These results highlight the nuanced decision-making dynamics that arise from the interaction between a customised robot and the type of suggestion. The longer reaction times in response to the “collect” suggestion in the customised condition suggest that participants may have experienced increased cognitive engagement and deliberation when interacting with a customised robot. These findings align with the notion that customisation in HRI can lead to increased engagement (Sung et al., 2007; Sung et al., 2009), encouraging users to consider their actions more carefully.

### 6.4 Perceived ownership

In line with our hypothesis (H2), our results demonstrated that customisation significantly enhances participants’ sense of ownership over the robot, which aligns with existing evidence on the endowment effect (Kahneman et al., 1990). This finding implies that customisation fosters a deeper, more personal connection with robotic systems, potentially influencing long-term engagement and acceptance.

### 6.5 Practical implications

These results highlight the complexity of customisation in HRI. The findings suggest that while customisation can enhance perceived ownership and influence decision times, its impact on compliance and risk-taking is less clear and likely context-dependent. These findings offer valuable insights into the design and deployment of robotic systems across various domains:

• Enhancing User Engagement through Customisation: Despite the lack of evidence supporting the impact of customisation on user compliance or risk-taking, the significant impact on perceived ownership and decision-making times presents a compelling case for incorporating customisation features in robot interaction design. For HRI developers and researchers, incorporating customisation in an interaction can enhance the user’s emotional and psychological investment in the system, potentially leading to improved long-term interaction and robot acceptance. Design options may include customising the robot’s appearance or adapting the robot’s behaviour to reflect the user’s preferences.

• Communication strategies: The persuasive power of robot suggestions, irrespective of customisation, highlights the importance of effective communication in robotic systems. For developers and researchers in HRI, understanding that customisation may not directly influence compliance but can affect user engagement and perception of the robot is crucial. This indicates that efforts to enhance robot-user interaction should focus more on the robot’s behaviour and the context of its suggestions rather than on physical customisation alone. Robots should be equipped with communication protocols that clearly explain the rationale behind suggestions and provide transparent information about their capabilities and limitations to enhance compliance and ensure users can make informed decisions. This understanding can inform the development of robotic support across various sectors, ensuring that they are both effective in their guidance and capable of fostering a positive user experience.

• Ethical Design and Implementations: Our findings reveal a general trust in robots and their potential to influence risk-taking behaviour regardless of customisation, in line with previous research (Hanoch et al., 2021), emphasising ethical considerations in designing robotic systems that foster trust and avoid over-reliance, especially in high-stakes and stressful situations where there is a higher likelihood of automation bias (Goddard et al., 2012). Developers must carefully balance the system’s persuasive capabilities with transparent communication about the robot’s limitations and the rationale behind its suggestions. Ensuring users have a realistic understanding of a robot’s functionality and decision-making process can mitigate overreliance and promote more informed user decisions. This includes implementing safeguards that prompt users to critically evaluate robot advice and incorporating feedback mechanisms that allow robots to learn from user interactions and adjust their behaviour to avoid promoting hazardous risk-taking.

Incorporating these considerations into HRI design and research can lead to more effective, ethically responsible robotic systems that respect user autonomy while providing meaningful assistance and engagement.

### 6.6 Limitations and recommendations for future research

Before embarking on this between-subjects study, a preliminary within-subjects investigation was conducted. This initial investigation shared a similar setup but with fewer customisation options and without recording data for inflates after help was requested. Initially, the presence of interaction effects led to concerns about the research method’s validity, resulting in the decision not to publish those findings. However, the consistency between the preliminary study’s outcomes and the results of the current investigation reinforces the validity of our initial observations, particularly regarding perceived ownership and risk-taking behaviour with the robot.

This iterative research process highlights the importance of further exploration in several key areas to deepen our understanding of robot customisation, trust dynamics, and their real-world applications:

1. Expand Diversity in Participant Demographics: Future studies should strive for a broader participant demographic beyond those with technical backgrounds. A more diverse participant pool, including variations in age, culture, and familiarity with technology, can provide a richer understanding of how different groups perceive and interact with customisable robots. This diversity will help in developing universally accessible and acceptable robotic systems.
2. User-Centric Design Research: Engaging users in the customisation process through participatory design research can unearth valuable insights into preferred customisation features and their impact on user experience. This approach can identify which aspects of customisation (e.g., aesthetic changes, behaviour adjustments) most significantly affect user engagement, trust, and compliance.
3. Longitudinal and Repetitive Testing: Customisation’s potential impact may not be fully captured by short-term interactions. By conducting longitudinal studies and utilising repetitive testing methods, we can evaluate both the stability and changes in the dynamics of user trust and rapport over time. This will provide a more comprehensive understanding of the long-term effects of customisation.
4. Exploration in Diverse Contexts: The study’s findings suggest that it is worth reconsidering the role of customisation in various Human-Robot Interaction (HRI) contexts, which may lead to more focused research in specific domains where customisation may play a more significant role. Future studies should expand beyond the BART and investigate how various decision-making tasks can benefit sectors such as healthcare, education, and assistive robotics. The BART measures the inclination to take risks while providing a standardised measurement that may not fully capture the intricacies of real-world decision-making processes in HRI. Additionally, the task did not effectively account for the influence of the total score and subjective priors of the robot’s performance and their eventual modification throughout the study (Strack and Deutsch, 2004) may have had a greater influence on decision-making processes than the effect of customisation.
5. Automation Bias and Decision Support: Given the evidence of automation bias in this study, future research should investigate strategies to mitigate overreliance on robot suggestions. This could involve designing robots that encourage critical thinking and decision-making skills, providing explanations for their suggestions, or highlighting the probabilistic nature of their advice. This research direction aligns with the need for a deeper investigation into human trust in robots, particularly in contexts where robots do not possess superior knowledge or capabilities. Understanding how environmental and task-related factors influence trust and reliance will contribute to designing ethical and supportive robotic aids.

Reflecting on the insights from both the preliminary study and aligning them with the findings from the current research, we pave the way for a comprehensive research agenda. This future research will not only validate the initial observations but also extend our understanding of human-robot interaction dynamics. The recommendations outlined here aim to address the nuanced complexities revealed through our studies, guiding the development of more ethical, user-friendly, and effective robotic systems that are attuned to the diverse needs and contexts of their human users.

## 7 Conclusion

This study set out to investigate the influence of robot customisation on decision-making and perception of robots. Our findings reveal that contrary to our hypothesis (H1), customisation did not significantly enhance compliance or risk-taking behaviours, indicating that in the context of this study, customising a robot does not inherently make it more persuasive. However, a significant effect of robot response on reaction time was observed, highlighting the nuanced role of interaction dynamics in decision-making processes. This result suggests that customisation, while not directly altering compliance or risk-taking tendencies, may enhance cognitive engagement and deliberation in scenarios where robots provide suggestions.

Moreover, consistent with our second hypothesis (H2), participants who customised the robot they used to complete the BART felt a stronger sense of ownership than those who did not customise. This finding emphasises the significance of customisation in forming deeper emotional connections between humans and robots, highlighting its role in enhancing the user experience beyond functional interaction.

Notably, the study underlines the importance of effective robot communication strategies and ethical design considerations, highlighting that user engagement and decision-making are influenced by customisation. The general trust in robot suggestions, irrespective of customisation, points to a potential automation bias, emphasising the need for a transparent and responsible robot design that encourages informed user decision-making.

In summary, our research indicates that while customisation does not directly affect compliance or alter risk-taking behaviour, it plays a critical role in enhancing perceived ownership and affecting decision-making times. Suggesting that while customisation enhances emotional and psychological engagement, its role in altering fundamental decision-making behaviours is limited and context-dependent. These insights contribute to the broader understanding of human-robot interaction and offer valuable considerations for designing and implementing personalised robotic systems.

Future research should investigate the nuanced effects of customisation in diverse HRI contexts and explore longitudinal impacts on user behaviour and perceptions. This study contributes to the growing body of HRI literature by providing a comprehensive analysis of the role of robot customisation, offering insights for developing more engaging, trustworthy, and ethically designed robotic systems.

## Data Availability

The raw data supporting the conclusions of this article will be made available by the authors, without undue reservation.
